# Exploring the Health Care Challenges and Health Care Needs of Arabic-Speaking Immigrants with Cardiovascular Disease in Australia

**DOI:** 10.3390/pharmacy7040151

**Published:** 2019-11-11

**Authors:** Erini Abdelmessih, Maree-Donna Simpson, Jennifer Cox, Yann Guisard

**Affiliations:** 1 School of Biomedical Sciences, Charles Sturt University, Leeds Parade, Orange 2800, Australia; masimpson@csu.edu.au (M.-D.S.); jcox@csu.edu.au (J.C.); 2 School of Science, Charles Sturt University, Leeds Parade, Orange 2800, Australia; yguisard@csu.edu.au

**Keywords:** Arabic, immigrants, cardiovascular, challenges, needs, preferences, unique, Australia

## Abstract

The Arabic-speaking immigrant group, which makes up the fourth largest language group in Australia, has a high prevalence of cardiovascular disease. The objective of this study was to explore the health care challenges and needs of Arabic-speaking immigrants with cardiovascular disease (CVD), using a comparative approach with English-speaking patients with CVD as the comparable group. **Methods:** Participants were recruited from community settings in Melbourne, Australia. Face-to-face semi-structured individual interviews were conducted at the recruitment sites. All interviews were audio-taped, transcribed, and coded thematically. **Results:** 29 participants with CVD were recruited; 15 Arabic-speaking and 14 English-speaking. Arabic-speaking immigrants, and to a lesser extent English-speaking patients with CVD may have specific health care challenges and needs. Arabic-speaking immigrants’ health care needs include: effective health care provider (HCP)-patient communication, accessible care, participation in decision-making, and empowerment. English-speaking participants viewed these needs as important for CVD management. However, only a few English-speaking participants cited these needs as unmet health care needs. **Conclusion:** This study suggests that Arabic-speaking immigrants with CVD may have unique needs including the need for privacy, effective HCP-patient communication that takes into account patients’ limited English proficiency, and pharmacist-physician collaboration. Therefore, there may be a need to identify a health care model that can address these patients’ health care challenges and needs. This, in turn, may improve their disease management and health outcomes.

## 1. Introduction

The burden of cardiovascular disease (CVD) is substantial in terms of its worldwide prevalence [1] and its health, social, and economic burden [2]. In 2016, 17.9 million people died from CVD globally [3]. The World Health Organization projects that by the year 2030, 23.6 million deaths will occur annually due to CVD [4]. In Australia, CVD is the second leading cause of disease burden, accounting for 15% of total disease burden. One person dies every 12 min due to CVD in Australia [5]. It is also a costly disease, as in 2012–2013 it consumed the largest proportion of health expenditure in Australia ($5.0 billion) [6].

Factors such as genetic predisposition, birthplace, country of origin, and culture have been shown to influence the prevalence of CVD and its risk factors between different ethnic groups (people who differ from a dominant group in cultural origin, religion, or colour) [7,8]. Several international studies [9,10,11] have suggested that Arabic-speaking immigrants (immigrants are people born overseas who have settled in a country permanently) [12] have a higher prevalence of CVD and CVD risk factors compared to many other ethnic groups, including the mainstream cultural group of the countries studied. In Australia, the Arabic-speaking immigrant group, which makes up the fourth largest language group (after English) [13], has a higher prevalence of CVD and recurrent CVD events compared to many other groups living in Australia [8,14,15]. It has been suggested that this may be due to lifestyle factors associated with their countries of origin, including the consumption of a high calorie diet, physical inactivity, and high smoking levels, which often persist in Australia [16].

The current arrival of immigrants/refugees from war-torn Arabic-speaking countries, who tend to have a higher rate of chronic diseases [17,18], may indicate that the prevalence of CVD among Arabic-speaking people in Australia is increasing substantially. 

There is a substantial risk of CVD event recurrence among patients with existing CVD [19]. An Australian study (Briffa 2011) showed that among a total of 28,941 participants with established coronary heart disease, 43% of participants had recurrent CVD events [19]. Additionally, there is a significantly higher risk of death due to a repeat CVD event, compared to an initial event [20]. CVD secondary prevention has been shown to decrease the risk of subsequent CVD events or death [21], improve functional status, and improve patients’ quality of life [22]. Therefore, it is suggested that one of the most effective ways of decreasing the burden of CVD is promoting CVD secondary prevention. International [21,23,24] and national [25] guidelines recommend CVD secondary prevention for all patients with existing CVD. Secondary prevention measures include: smoking cessation, low alcohol consumption, being physically active, weight management, blood pressure control, type 2 diabetes mellitus management, depression screening and treatment, using pharmacotherapy, and secondary prevention programs including cardiac rehabilitation [21].

There is good evidence to suggest that immigrants face many challenges to secondary prevention compared to their non-immigrant counterparts [26]. These challenges include poor access to secondary prevention services [27], and challenges to self-management [28,29]. 

Patients with CVD need to self-manage their disease on a daily basis, by managing their symptoms, adhering to pharmacotherapy, and adopting a healthy lifestyle [30]. Despite the benefits associated with self-management, particularly in the prevention of recurrent CVD events, patients of ethnic backgrounds may have fewer resources to enable them to self-manage their chronic disease [28,29]. For example, it has been reported that they are usually not provided with sufficient information regarding appropriate medication use [28]. Furthermore, few disease self-management interventions have been tailored to enable their application to be adapted to ethnic minority patients [31,32]. 

Poor understanding of these immigrants’ health care challenges and needs may contribute to poor self-management as patients may not be sufficiently equipped to manage their own health [33], which may increase their risk of recurrent CVD events or death [26]. There is a paucity of studies regarding the challenges facing Arabic-speaking immigrants with CVD worldwide, and their needs to be able to ideally manage their CVD. Therefore, the aim of this study was to explore the health care challenges and needs of Arabic-speaking immigrants with CVD, using a comparative approach whereby English-speaking patients with CVD were used as the comparable group. Comparing Arabic-speaking immigrants’ health care challenges and needs to those of English-speaking patients with CVD will help to determine whether a health care provider’s approach to assisting Arabic-speaking immigrants to manage their CVD needs to be a tailored approach, or, whether it could be a common approach for both patient groups. 

## 2. Materials and Methods 

### 2.1. Research Design

This study was approved by the Charles Sturt University Human Research Ethics Committee (protocol number 400/2017/10). The study was conducted between March and June 2018.

A qualitative approach involving semi-structured individual interviews was used. To gain an in-depth understanding of participants’ health care challenges and needs, an interview guide consisting of open-ended questions was developed following an extensive literature review. Questions were refined during the course of the interviews. A semi-structured interview schedule allowed participants to speak freely, yet allowed for exploration of the specific issues that were intended to be explored [34]. A written informed consent form was signed by each participant before starting the interview.

### 2.2. Recruitment and Setting

Purposive sampling was used in this study, to ensure recruitment of participants with a range of socio-demographic characteristics, disease characteristics, and experiences with cardiovascular disease management. This sought to include all significant points of view in this study. Further, a snowballing sampling strategy was used whereby participants were asked to refer other patients who fit the eligibility criteria. Participants were eligible to be included in this study if they met certain inclusion and exclusion criteria (Table 1). 

Participants were recruited through two community centers, four senior citizens clubs, and one general health practice in metropolitan locations in Melbourne, Australia. Patients were informed about the study by each recruitment site’s receptionist. If potentially eligible patients expressed interest in participating in the study, they were referred to the student researcher for more details about the study. Potential participants who indicated that they may be willing to take part in the research were screened for eligibility. If eligible, potential participants were provided with written information regarding the project, and a consent form. Twenty-nine interviews (15 Arabic-speaking participants, and 14 English-speaking participants) were conducted at the recruitment sites. 

### 2.3. Data Collection and Analysis 

Individual interviews rather than focus group interviews were used in this study as Arabic-speaking people tend to value privacy [35]. Each interview lasted between 45 and 55 min. Demographic information was collected from each participant before the interview, using a brief survey. During the interview, participants were asked about the health care challenges that they face when managing their CVD and their needs to be able to address their health care challenges. Participants were asked questions about their knowledge regarding secondary prevention, the secondary prevention measures they undertake, their barriers to secondary prevention, and their perceptions regarding HCPs. For Arabic-speaking participants, the interview guide was available in Arabic as well as English, to suit participant preference. However, all Arabic-speaking participants indicated a preference to be interviewed in English.

The interviews were audio recorded and then transcribed verbatim. Hand-written notes about the interviewer’s observations during interviews were also noted. All transcripts were checked against the original audio recordings to ensure precision. 

Interviews continued to be conducted until data saturation was attained. That is, when no new information or themes arose. 

Before analysing the data, the first author created a draft of the theory driven codes, which was then reviewed by another three researchers (the student researcher’s research supervisors). Then, all researchers finalised the theory driven code book. The collected data was imported into NVivo software (QSR NUD Vivo: version 11) and organised into themes that described participants’ views using a thematic analysis framework. The thematic analysis approach which is a commonly used qualitative analytical approach was used to analyse the data for the following reasons. Firstly, thematic analysis concentrates on finding meaning patterns across participants, to obtain more generalized views [36]. Secondly, it is flexible, allowing the provision of a rich and detailed account of the data. Thirdly, it is useful for analysing data suited to inform interventions. This study’s aim was to explore participants’ health care challenges and needs, and use the findings to inform the implementation of a health care model to address these health care challenges and needs [37]. 

For thematic analysis of the data, the data was read line-by-line to determine the various responses for each interview guide question. Following this step, the data was coded for identification of important findings, and then organised into themes that described the perceptions and views of participants. While some codes were informed by the theory driven codebook, new data driven codes were also developed during analysis. Participant quotations were selected to clarify the creation of various themes and subthemes. Relationships between the coded data and participants’ demographic information were explored. 

### 2.4. Rigor

This study followed Guba and Lincoln’s standards for transferability, credibility, and confirmability [38]. To establish credibility, the steps taken to analyse the data have been discussed. Additionally, participants’ responses were recorded word for word, and transcribed word for word without any changes. Credibility was also attained by choosing research methodology and techniques that suited the research question. To enhance transferability, a thick description of the methodology and research procedures used was provided. This will allow the reader to assess their transferability and potential to be implemented in other settings. However, this study’s sample size of 29 participants (like many other qualitative studies) is not large enough to enable generalization. In order to establish confirmability, analyst triangulation (that is, using different analysts to review the findings) was used. 

## 3. Results

### 3.1. Study Participants 

The sociodemographic characteristics of participants are shown in Table 2. The two groups of participants were of a similar age, similar female to male ratio, similar education status, and similar employment status. Compared to English-speaking participants, more Arabic-speaking participants were living with family, and rated their health as unsatisfactory to poor.

While some Arabic-speaking participants had poor English-speaking proficiency, they were able to express their views in English clearly.

### 3.2. Themes

Participants’ perceptions and point of view regarding their health care challenges and needs revolved around six themes, being: Exploration of the biological and social aspects of the disease by health care providers (HCPs), effective communication with patients, accessible care, participation in decision-making, patient empowerment, and the role of the pharmacist.

#### 3.2.1. Exploration of the Biological and Social Aspects of the Disease by Health Care Providers 

##### Biological Aspect

Most Arabic-speaking participants reported that their HCPs do not explore all their health-related issues or address them. Many reported that HCPs only allow them to discuss one health- related issue/concern during any one consultation or pharmacy visit. These issues appeared to be more pronounced among respondents with poor English-speaking skills, who visit English-speaking HCPs.

Most Arabic-speaking participants reported that HCPs do not explore each patient’s unique health care needs and preferences, with some attributing this to stereotyping.

In contrast, most English-speaking participants reported that their HCPs explore all their health-related issues and address them, and encourage them to express their health concerns. 

##### Social Aspect

In contrast to English-speaking participants’ views, most Arabic-speaking participants indicated that HCPs should not explore patients’ social context including family, cultural and religious beliefs, and employment. In the view of some participants, exploring these issues is a violation of the patient’s privacy, while for others it is a cause of discrimination/racism as they may be perceived as different.

A few Arabic-speaking participants encountered racism within the health care system. One participant recalled that her culture was mocked by HCPs in hospital, which resulted in her family’s poor trust in the health care system, and their reluctance to seek health care.

*“Staff [hospital staff] made fun of my culture there … so we [including family members] do not want to go back there.” [Arabic-speaking participant 3]*

#### 3.2.2. Effective Communication with Patients

Most Arabic-speaking participants reported that their HCPs are unapproachable, as they do not listen to them, and do not encourage them to express their health concerns. Many participants cited lack of time as a possible reason for HCPs’ unapproachability. Unapproachability appeared to be disclosed more by Arabic-speaking participants who visit English-speaking HCPs, particularly those with poor English-speaking skills.

*“If I speak honest, they haven’t time to listen to me … no time. He tried to finish.” [Arabic-speaking participant 7]*

Many Arabic-speaking participants reported that their HCPs are not clear as they do not use simple language, tend to speak fast, and do not provide them with clear information regarding their health condition, treatment plan, and medications. They suggested that this impacts on their ability to manage their disease.

*“I think he must speak slowly because … because I cannot understand if he speaks to me normal. He can explain it in easy language not medical language or I can’t know how to take medicine the right way.” [Arabic-speaking participant 7]*

Participants who indicated that they prefer using an interpreter have reported that they do not get offered an interpreter service.

A few English-speaking participants reported that their HCPs are unapproachable, as they do not listen to them. Additionally, a few participants reported that their HCPs are not clear as they do not provide them with clear information and tend to use medical terms.

*“Because of time limits, they tend to be unclear. For example, my pharmacist does not explain much.” [English-speaking participant 6]*

English-speaking participants indicated that clarity is important in self-management. 

#### 3.2.3. Accessible Care

Almost all Arabic-speaking participants reported that they face access issues. The most cited access issue by Arabic-speaking participants was poor access to information regarding their health condition, treatment plan, and medications, particularly by pharmacists.

*“They [HCPs] do not give enough information like about medicine or about how I’m going with health … I think important to tell me how I’m going or how to use my medicine, things like that.” [Arabic-speaking participant 13]*

Many Arabic-speaking participants preferred written information to enable them to read the information more carefully at home to comprehend it, or to enable their children to translate for them. However, some Arabic-speaking participants preferred verbal information as they are illiterate (in both languages or English only). 

Almost all Arabic-speaking participants identified that they do not need written information that is tailored to their cultural needs as individuals’ cultural needs vary, particularly that different Arabic countries have slightly different cultures, and different parts of the same country may have slightly different cultures. 

A few English-speaking participants reported that they face access issues including poor information provision, particularly by pharmacists. Many participants indicated that they try to address their access issues such as poor information access.

*“I do not recall getting any written information about any medication. I was never asked whether I was going well with my heart medications … I do my own research.” [English-speaking participant 5]*

Most English-speaking participants indicated that they prefer written information to enable them to read the information more carefully at home, or to keep it for future reference. A few participants indicated that they prefer electronic information (websites and emailed information). These participants tended to be young and held a university degree.

#### 3.2.4. Patient Empowerment

English-speaking participants appeared to have more knowledge regarding CVD secondary prevention than Arabic-speaking participants. Further, most English-speaking participants reported that they were undertaking secondary prevention measures. 

Most Arabic-speaking participants reported that they follow a healthy diet plan. A few participants reported that a healthy diet caused their health to deteriorate.

*“He [general practitioner] tell me go home open the fridge, throw all the bread, cake, rice, macaroni, spaghetti … Throw them away. I followed that for a week but I could not follow. The symptoms getting worse when I follow that [diet].” [Arabic-speaking participant 5]*

While a few Arabic-speaking participants reported that they exercise, others reported that they do not exercise due to the perception that it worsens their condition, or the belief that their health is under God’s control. 

When asked whether they would be interested in joining CVD-related health programs to empower them to undertake secondary prevention measures, some Arabic-speaking participants expressed interest. However, only a few Arabic-speaking participants have been informed of CVD-related health programs. Participants who indicated that they would not be interested in joining these programs provided reasons including poor English proficiency, and belief in fate. 

Unlike Arabic-speaking participants, almost all English-speaking participants have been informed of CVD-related health programs, which many viewed as important in self-management. 

When asked whether HCPs empower them to self-manage their disease, more than half of the Arabic-speaking participants reported that their HCPs do not empower them to self-manage their disease. Participants provided reasons including HCPs’ lack of time. 

In contrast, many English-speaking participants reported that their HCPs empower them to self-manage their disease. 

#### 3.2.5. Participation in Decision-Making

Unlike English-speaking participants, most Arabic-speaking participants indicated that they prefer not to be involved when health care decisions are being made regarding their health. Most of those participants perceived they had poor health literacy, and therefore were concerned that they may make the wrong health care decisions.

*“I leave up to them. I am not a doctor and do not know so I cannot make decision. I leave up to them … otherwise I can damage health.” [Arabic-speaking participant 1]*

It should be noted that more than half of the Arabic-speaking participants reported that when they do not agree with their HCPs’ advice, they do not follow it. 

#### 3.2.6. Role of Pharmacists

Both participant groups indicated that they have higher trust in physicians’ expertise than pharmacists’ expertise. 

Most Arabic-speaking participants reported that their pharmacists dispense their medications and, at times, counsel them regarding new medications. However, they do not provide them with sufficient information (including directions for use, and side effects), do not follow up regarding ongoing medications, and do not explore, listen to, or address their medication-related concerns, or, answer their questions, with some citing pharmacists’ lack of time as a possible reason. Many perceived that pharmacists regard themselves as “business owners”, not health professionals who look after patients’ health care needs. 

Arabic-speaking participants indicated that they have significant trust in pharmacists in the Arabic-speaking countries. They discussed the extensive disease management role these pharmacists have, suggesting that this impacts on patients’ trust in pharmacists. 

Arabic-speaking participants indicated that they would be keen to join pharmacy-based cardiovascular disease management services. Participants suggested that the care provided by pharmacists in these services may complement the limited care provided by physicians (due to physicians’ lack of time), and may prevent recurrent CVD events.

*“For sure. Doctor have no time to do all check for heart. If pharmacist can do that, is good because I check all time and I am relax, and not have another heart attack.” [Arabic-speaking participant 12]*

A few English-speaking participants reported that pharmacists do not provide them with their needs, including the need for information. 

English-speaking participants viewed pharmacists’ role as more limited than that viewed by Arabic-speaking participants, as they suggested that pharmacists’ roles should only include dispensing and patient counselling. Most English-speaking participants identified that they would not be willing to join pharmacy-based cardiovascular disease management services, providing reasons including inconvenience.

## 4. Discussion

Previous studies found that Arabic-speaking immigrants worldwide have some health care challenges and needs, particularly the need for cultural sensitivity towards issues such as modesty [16]. Studies emphasized physicians’ role in addressing these issues [16,39]. This study adds to earlier work by exploring CVD patients’ perspective, in the Australian context. The study’s results also yielded new knowledge regarding participants’ health care challenges and needs, and how cultural sensitivity is rejected by this group due to fear of being stereotyped. 

### 4.1. Effective Communication with Patients

Both groups expressed the view that having effective communication skills, which entail clarity, approachability, and two-way communication is important in promoting appropriate use of medicine, self-management, and treatment adherence. This is consistent with the findings of previous research [40,41,42].

Poor HCP-patient communication was reported by most Arabic-speaking participants, and a few English-speaking participants. This is in line with the findings of previous research which shows that poor HCP-patient communication tends to be more prominent among immigrants [42]. This is due to these patients’ poor English-speaking skills, which may contribute to their inability to understand HCPs, express their health concerns, and discuss symptoms, leading to negative health- related consequences including misdiagnosis [43]. However, participants who have stressed the need for interpreter services to be present during medical encounters have indicated that this service is never offered to them. This is consistent with previous research which shows that only 50% of HCPs offer interpreting services to patients with limited English [44]. This is despite the availability of free interpreting services, covering over 160 languages [45].

Other barriers to effective HCP-patient communication cited by Arabic-speaking participants were physicians’ use of medical terms (which was also cited by a few English-speaking participants), and the fast speed with which many HCPs speak. These barriers were also cited by participants from non-English speaking backgrounds in a Sydney-based study (Garrett 2008). According to the study, these communication barriers contributed to poor care provision, and poor compliance [40]. Therefore, there may be a need for HCPs to provide patients with simplified information that suits their needs and ensure that they understand this information.

Lack of HCPs’ time has been cited as a reason for poor HCP-patient communication and lack of information access in this study, and in other studies [28]. While physicians’ short consultation time is determined by Medicare, the HCP could plan multiple consultations with the patient until they are both satisfied that the patient’s health concerns are identified and addressed. Additionally, patient referral to home medication reviews (HMR) or Medscheck may allow pharmacists to spend enough time with each patient, to explain treatments, answer the patient’s questions, and ensure safe use of medications.

### 4.2. Accessible Care 

The most common access barrier cited by both participant groups was poor health information access. Participants spoke at length about poor information provision, particularly by pharmacists. This finding is consistent with findings of previous research [28,46]. In a study by Girgis and Ward (Girgis and Ward 2004), only 19.1% of participants (Arabic-speaking patients with diabetes) reported receiving diabetes-related information from their HCPs in the previous year [46]. Information access may enhance patients’ understanding of their disease and treatment, which in turn may enhance adherence, self-management [47], and health outcomes [48].

It was observed in this study that the type of information needed and the information modalities preferred by various patients with the same condition (even within the same cohort) varied depending on factors including English proficiency, and literacy/health literacy level; pointing to the need for a patient-centered care approach to information provision. Pharmacy services such as HMR and pharmacy-based chronic disease monitoring services may enable pharmacists to play a major role in the provision of patient-centered information access, by providing patients with medication- related information that suits each patient’s information needs, using a tailored style that suits each patient’s needs, and, by following up on ongoing medication use. This may improve these patients’ use of medicine, enhance self-management, and improve health outcomes.

### 4.3. Patient Empowerment

Arabic-speaking participants appeared to have poor knowledge regarding CVD secondary prevention and some Arabic-speaking participants have inaccurate beliefs regarding secondary prevention measures [39,49]. These issues may possibly be due to having poor health literacy, poor access to health information, and lack of empowerment by HCPs. Despite Arabic-speaking participants’ need to be empowered regarding CVD secondary prevention, many participants have indicated that their HCPs do not empower them to undertake appropriate secondary prevention measures. Empowerment may be important for those with CVD as it may enhance secondary prevention, health outcomes, and decrease health service use [50]. This highlights the importance of patient referral to health programs, which may empower patients to appropriately manage their disease, given HCPs’ short consultation time. 

### 4.4. Exploration of the Social Aspect of the Disease by Health Care Providers 

In contrast to English-speaking participants’ views, most Arabic-speaking participants regarded exploring patients’ social context (including employment, and cultural and religious beliefs) as a violation of patients’ privacy, which is highly valued in their culture [51]. Studies conducted worldwide show that Arabic-speaking immigrants’ health seeking behavior is determined by whether HCPs and health programs use strategies to maintain patients’ privacy [52]. Therefore, it may be necessary for HCPs to explain to patients the reason for asking any personal questions, before gathering any personal information, and, to assure them that their privacy will be maintained. 

Arabic-speaking participants perceived consideration of their cultural/religious beliefs, and being provided with tailored health information, as being regarded as different (i.e., not being regarded as members of the Australian society). Participants did not prefer to be regarded as different and preferred to be viewed as integrated members of the Australian society to avoid racism, which some have reported experiencing. According to Jackson and Yoo (2012), there is a positive relationship between lack of integration in a host society and “perceived racism”. Participants who reported that they experienced racism have indicated that it resulted in distrust of the health care system. Distrust of the system may have an impact on Arabic-speaking patients’ health, and health seeking attitudes [53].

### 4.5. Participation in Decision-Making

Unlike English-speaking participants, most Arabic-speaking participants do not prefer to be involved when health care decisions are being made regarding their health. This difference between the two groups may be due to Arabic-speaking participants’ perception that they have poor health literacy, their cultural barriers to involvement (including their high regard and respect for physicians’ opinions) [35,54], and the significant HCP-patient communication barriers that Arabic-speaking participants face. 

It appears that there is a misunderstanding among Arabic-speaking participants regarding what involvement in decisions entails, as they perceive that it involves patients making their own health care decisions. Given that most of these participants viewed themselves as having poor health literacy, they preferred not to be involved. This finding is consistent with the findings of previous research which shows that poor health literacy is a significant barrier to shared decision-making [55,56]. Therefore, it is proposed that Arabic-speaking immigrants’ understanding regarding involvement could be rectified through workshops which could be conducted at Arabic community centres, mosques, and churches. 

Involvement may be particularly important for Arabic-speaking patients, as many participants reported that they do not adhere to advice that they do not agree with. Additionally, some Arabic-speaking people’s cultural/religious beliefs may affect the type of treatment that they prefer, and in turn may affect treatment adherence. Involvement may also enhance HCPs’ ability to determine patients’ concerns and needs [57], which, according to this study’s participants, are not explored or addressed, which led to significant negative health outcomes (recurrent CVD events) in some cases. 

### 4.6. Role of the Pharmacist

In this study, both participant groups indicated that they have higher trust in physicians’ expertise than pharmacists’ expertise. It is possible that pharmacists’ lack of information provision, and lack of patients’ awareness regarding pharmacists’ extended role in Australia (including chronic disease management) may have given participants the perception that pharmacists are not as competent HCPs as physicians [58]. Further, many participants perceived that pharmacists’ main motive may be financial gain, which may have further contributed to participants’ poor trust in pharmacists compared to physicians [58]. 

Arabic-speaking participants indicated that they have significant trust in pharmacists practicing in Arabic-speaking countries. In Arabic-speaking countries, where most patients tend to be of low socioeconomic status, patients tend to rely on pharmacists to prescribe and dispense medications, and manage their disease as they tend to be unable to pay for a physician’s consultation [59]. Therefore, participants’ awareness of the extensive role that pharmacists in the Arabic-speaking countries have, and their frequent encounters with these pharmacists, may have led to the development of a good HCP-patient relationship that is characterised by trust, particularly when provided with a good quality service. Trust in HCPs has been shown to promote adherence [60] and health seeking behavior [61], which are important in secondary prevention.

Pharmacy-based chronic disease management services may improve patients’ trust in pharmacists’ expertise, as pharmacists may have sufficient time to assist patients in managing their disease, listen to their concerns, and address them accordingly. These, in turn, may improve secondary prevention and health outcomes.

While Arabic-speaking participants indicated that they would be keen to join pharmacy-based cardiovascular disease management services, about half of the participants indicated that they would be interested in joining other CVD-related health programs, such as rehabilitation programs. This may be because Arabic-speaking participants reported poor access to medication-related information (verbal and written). Therefore, they may have all expressed more interest in pharmacy-related programs to ensure that they receive sufficient medication-related information from the pharmacist, who may be obligated to spend sufficient time with them as part of the program.

According to Arabic-speaking participants, pharmacy-based disease management services promote pharmacist-physician collaboration which may prevent recurrent CVD events and improve health outcomes, particularly given physicians’ short consultation times. Studies showed that pharmacy-based disease management services promote adherence [62] and improve health outcomes [63]. Despite these benefits, these services are currently under-used in preventative care [64].

## 5. Strengths and Limitations of the Study

Limitations of this study include the use of purposive sampling and the small sample size, which may hinder the generalisation of the findings without further research. Qualitative research is concerned with explaining a phenomenon rather than examining statistical generalisation across a certain population [65]. Hence, this study’s recruitment method aimed at recruiting participants with maximum variation to be able to obtain rich data, which is a strength of this study.

The only Australian states that are heavily populated with Arabic-speaking people are NSW and Victoria. A limitation to this study is that no interviews were conducted in NSW. This was due to the high financial cost associated with conducting the interviews in NSW. Therefore, sampling bias is possible. However, given that the inclusion and exclusion criteria would not have changed if the interviews were conducted in NSW, it is anticipated that the responses may not have varied significantly between the two states.

## 6. Impact on Policy and Practice

From a policy perspective, this study sheds light on the need to address the range of health care challenges and needs of Arabic-speaking immigrants with CVD, a fast-growing community with high prevalence of CVD. These challenges seem closely linked to illiteracy, poor English proficiency, and poor health literacy levels. These manifest as low levels of adherence to secondary prevention measures, and a poor HCP-patient relationship, particularly the pharmacist-patient relationship. There is a need to raise awareness regarding pharmacists’ significant roles in disease management. This may enhance pharmacists’ practice capacity to manage these patients. Additionally, there is a need for Community Pharmacy Agreements regarding pharmacy funding, which may enable outreach to this patient group.

## 7. Conclusions

This study has indicated that Arabic-speaking participants have a wider range of health care needs and challenges than English-speaking participants with CVD. Arabic-speaking immigrants’ health care needs include: accessible care, effective HCP-patient communication, and patient empowerment. These needs were viewed by English-speaking participants as important in CVD management. However, only a few English-speaking participants cited these health care needs as unmet needs. It was suggested that Arabic-speaking participants may have unique health care needs, including the need for assured privacy, effective HCP-patient communication that is tailored to their needs based on English proficiency and health literacy, and the need for pharmacist-physician collaboration via interventions such as pharmacy-based CVD management services. However, given the small sample size in this study, further investigation is warranted.

There may be a need to identify a health care model that can address these patients’ health care challenges and needs. This, in turn, may improve their disease management, prevent recurrent CVD events, and improve their health outcomes.

## Figures and Tables

**Table 1:** Study inclusion and exclusion criteria.

Inclusion Criteria	Exclusion Criteria
Arabic-speaking participants:	Arabic-speaking participants:
Eligible patients were 18 years of age or older. Eligible patients were born in an overseas country that speaks Arabic, and their first language is Arabic. Eligible patients must have been diagnosed with cardiovascular disease at least six months ago.	Patients with cognitive impairment.
English-speaking participants:	English-speaking participants:
Eligible patients were 18 years of age or older. Eligible patients were self-identified as English-speaking, which is defined as someone whose first language is English, born in North America, Australia, the United Kingdom, or New Zealand. Eligible patients must have been diagnosed with cardiovascular disease, at least six months ago.	Patients with cognitive impairment.

**Table 2:** Participants’ sociodemographic characteristics (n = 29).

Characteristic	Arabic-Speaking Patients with CVD	English-Speaking Patients with CVD
Gender:		
-Female	8 (53.0%)	8 (57.1)
-Male	7 (47.0%)	6 (42.9)
Age		
40–49	3 (20.0%)	2 (14.3%)
50–59	3 (20.0%)	3 (21.4%)
60–69	4 (26.7%)	2 (14.3%)
70–79	4 (26.7%)	5 (35.7%)
80–89	1 (6.7% )	2 (14.3%)
Average number of years in Australia	25.8 years (3–57)	N/A
Education status		
-Primary school education	2 (13.3%)	2 (14.3%)
-High school education	8 (53.3%)	7 (50.0%)
-University degree or above	5 (33.3%)	5 (35.7%)
Employment status		
-Employed	5 (33.3%)	5 (35.7%)
-House wife	3 (20.0%)	Nil
-Unemployed	2 (13.3%)	2 (14.3%)
-Retired	5 (33.3%)	7 (50.0%)
Living arrangement		
-Living with family	12 (80.0%)	7 (50.0%)
-Living alone	3 (20.0%)	7 (50.0%)
English proficiency (self-rated)		N/A
-Poor	3 (20.0%)	
-Unsatisfactory	3 (20.0%)	
-Satisfactory	6 (40.0%)	
-Excellent	3 (20.0%)	
Health status (self-rated)		
-Poor	3 (20.0%)	Nil
-Unsatisfactory	7 (46.7%)	5 (35.7%)
-Satisfactory	4 (26.7%)	7 (50.0%)
-Excellent	1 (6.7%)	2 (14.3%)
Country of origin		
-Egypt	6 (40.0%)	N/A
-Sudan	3 (20.0%)	
-Lebanon	2 (13.3%)	
-Syria	2 (13.3%)	
-Iraq	1 (6.7%)	
-Palestine	1 (6.7%)	
